# Vaccination Against Diphtheria and Tetanus as a Way to Activate Adaptive Immunity in Children with Solid Tumors

**DOI:** 10.3389/fimmu.2021.696816

**Published:** 2021-07-08

**Authors:** Mikhail Petrovich Kostinov, Nelli Kimovna Akhmatova, Svetlana Victorovna Karpocheva, Anna Egorovna Vlasenko, Valentina Borisovna Polishchuk, Anton Mikhailovich Kostinov

**Affiliations:** ^1^ Department of Allergology, I.I. Mechnikov Research Institute of Vaccines and Sera, Moscow, Russia; ^2^ Department of Epidemiology and Modern Vaccination Technologies, Sechenov First Moscow State Medical University, Moscow, Russia; ^3^ Department of Immunology, I.I. Mechnikov Research Institute of Vaccines and Sera, Moscow, Russia; ^4^ Department of Medical Cybernetics and Computer Science Novokuznetsk State Institute for Advanced Training of Physicians, Branch Campus of the Russian Medical Academy of Continuous Professional Education, Novokuznetsk, Russia

**Keywords:** solid tumors, vaccination in children, diphtheria and tetanus toxoid, T-lymphocytes, post-vaccination antibodies

## Abstract

Early studies on vaccination of children with oncological diseases were only dedicated to the assessment of safety and immunogenicity of the drug. Mechanisms of the post-vaccination immune response were not investigated. This study involved 41 patients aged 7-15 years who were treated for solid tumors two or more years ago. Of these, 26 were vaccinated against diphtheria and tetanus with ADS-m toxoid. Fifteen children (i.e., controls) were not vaccinated. The vaccination tolerability and clinical characteristics of the underlying disease remission ware assessed. Lymphocyte subpopulations were investigated over time by flow cytometry at 1, 6, and 12 months. IgG anti-diphtheria and anti-tetanus toxoids levels were assessed by ELISA. Within the first day of the post-vaccination period, two (7.7%) children demonstrated moderate local reactions and increased body temperature (up to 38.0°C). Relapse and metastasis were not mentioned within a year after immunization. An increase in concentration of IgG antibodies, maintained for 12 months, were noted [2.1 (1.3-3.4) IU/ml against diphtheria (p <0.001), 6.4 (2.3-9.7) IU/ml against tetanus (p <0.001)]. In contrast to healthy children, those with a history of cancer demonstrated a decrease in the relative number of mature T lymphocytes, as well as in absolute number of cytotoxic T cells and B lymphocytes. In a month after the revaccination, a significant increase in absolute (p = 0.04) and relative (p = 0.007) numbers of T lymphocytes and T helpers was revealed. In a year, these values decreased to baseline levels. As for helpers, they decreased below baseline and control values (p = 0.004). In a year after the vaccination, there was a significant (p = 0.05) increase in lymphocyte level with a decrease in the number of NK cells and B cells as compared with controls. Revaccination against diphtheria and tetanus promoted proliferation of a total lymphocytic cell pool along with restoration of the T lymphocyte subpopulation in children with a history of solid tumors. The ADS-m toxoid has a certain nonspecific immunomodulatory effect. These findings are important, also in the midst of the coronavirus pandemic.

## Introduction

After more than 200 years of development and application, vaccination has been recognized as the most efficient and mandatory measure to combat infections. In studies focused, mainly, on specific antibody formation and their level of maintenance duration, clinical tolerability, and epidemiological effects of the vaccination, other immune mechanisms that occur in a vaccinated individual upon administration of an immunobiological drug remain undisclosed. The fact that such changes in immune response exist indisputably is evidenced not only by synthesis of post-vaccination antibodies, but also by positive clinical effects in vaccinated patients [including a decreased incidence of secondary non-vaccine preventable infections, as well as longer remission in patients with concomitant chronic diseases (1–4)].

All of the abovementioned facts mean that development of approaches to model the number and quality of T cells before, during, and after various treatments is of interest (5).

Currently, a relationship of immune stimulation with administered heterologous vaccines (for example, ones against measles and BCG) is mentioned. Immunization enhances the body’s response to other pathogens that are not contained in the vaccine. In turn, this leads to decreased child mortality (6). While a nonspecific effect of many vaccines against influenza, pneumococcal infection, measles, and tuberculosis has been reported, a similar effect of diphtheria and tetanus vaccines is understudied.

The study objective was to investigate an immunoregulatory effect of vaccination with diphtheria and tetanus toxoids on adaptive immunity parameters in children with a history of solid tumors.

## Materials

### Clinical Study Design

The main aims of the study were to evaluate an effect of a vaccine against diphtheria and tetanus on regeneration of T lymphocytes in 26 patients with solid tumors treated two or more years ago, to assess an ability to form specific antibodies and to characterize clinical tolerability of the vaccination in the light of local and systemic reactions at an early date (1-30 days). Secondary tasks were to investigate parameters of adaptive immunity over time along with assessment of the lymphocyte subpopulation and levels of serum antitoxic antibodies against diphtheria and tetanus within 6-12 months, as well as to study the duration of the underlying disease remission after vaccine administration.

The study included a control group of 15 children with a history of solid tumors who did not receive vaccination against diphtheria and tetanus during remission. Clinical aspects of remission and subpopulation of lymphocytes were assessed over time.

A phase IV (post-marketing) non-randomized controlled study was conducted in two centers in Moscow (Russia). The protocol was approved by the local Ethics Committee of the Federal State Budgetary Institution Blokhin National Medical Research Center of Oncology of the Ministry of Healthcare of the Russian Federation. The study was carried out in accordance with the Declaration of Helsinki, the Guidelines of the International Council for Harmonization for Good Clinical Practice and Russian regulations. Written informed consent was obtained from parents before enrollment of their children in the study.

### Clinical Laboratory Examination

Children of the main group who were to be immunized and controls underwent a comprehensive examination in the Research Institute of Children Oncology and Hematology Federal State Budgetary Institution Blokhin National Medical Research Center of Oncology of the Ministry of Healthcare of the Russian Federation. The examination included examination by a pediatric oncologist; US of abdominal organs and retroperitoneal space (according to CT/MRI); computed tomography of chest organs; radioisotopic test (if medically required); investigation of biological tumor marker levels (NCE - neuron-specific enolase, AFP - alpha fetoprotein, hCG - human chorionic gonadotropin, etc.); blood chemistry; general blood test with platelet level investigation; determination of a number of lymphocytes and their subpopulations, as well as post-vaccination levels of antibodies against diphtheria and tetanus.

Twenty-six children without relapse and metastasis signs were recommended to receive a vaccination against diphtheria and tetanus.

### Vaccinated Children Follow-up

The vaccinated children underwent an in-depth clinical observation by a pediatric oncologist for 12 months and more. It included a comprehensive examination of the underlying disease; investigation of immune status and serological examination to detect antitoxic antibodies against diphtheria and tetanus at 1, 6, 12 and more months after the vaccination. Controls (i.e., unvaccinated children) underwent the same comprehensive examination of the underlying disease at the same time (with the exception of a reading of the level of post-vaccination antibodies).

Blood sampling and vaccination was carried out in compliance with all the aseptic and antiseptic regulations using disposable instruments and sterile test tubes in a treatment room of the Research Institute of Children Oncology and Hematology Federal State Budgetary Institution Blokhin National Medical Research Center of Oncology of the Ministry of Healthcare of the Russian Federation and the Federal State Budgetary Scientific Institution Mechnikov Research Institute of Vaccines and Sera.

### Disease History and Status

According to **Table 1**, most children (17 [41%]) suffered from nephroblastoma. Neuroblastoma and soft tissue sarcoma was diagnosed in 5 (12%) and 7 (17%) patients, respectively. Germ cell tumors were mentioned in 4 (10%) children, and 8 children (20%) had other oncological nosological forms (retinoblastoma - 2, osteosarcoma - 2, melanoma - 2, hepatoblastoma - 1, Hodgkin’s lymphoma - 1). According to the distribution of patients by nosological forms, the study groups were comparable (p = 0.98).

**Table 1:** Nosological forms of diseases in revaccinated children (n = 26) and controls without revaccination (n = 15).

Nosological forms	Total		Study groups			
			Vaccinated persons		Controls	
	Abs.	%	Abs.	%	Abs.	%
Nephroblastoma	17	41%	11	42%	6	40%
Neuroblastoma	5	12%	3	12%	2	13%
Soft tissue sarcomas	7	17%	4	15%	3	20%
Germ cell tumors	4	10%	3	12%	1	7%
Other	8	20%	5	19%	3	20%
Total	41	100%	26	100%	15	100%

### Vaccinal Status

Revaccination with the ADS-M toxoid was carried out in 26 children during remission (>2 years). Patients already had a history of 2-3 vaccinations against diphtheria, tetanus, and pertussis before the onset of an oncological disease. Children (16 boys [61%] and 10 girls [39%]) were aged 7-15 years (11 ± 2.4 years).

Controls (n = 15) also had a history of an incomplete primary course of vaccination against diphtheria, tetanus, and pertussis. Later, during remission (>2 years), they were not revaccinated due to a possible adverse effect of the immunization on carcinoma recurrence. Children (10 boys [67%] and 5 girls [33%]) were aged 7-15 years (10 ± 2.9 years).

Revaccinated children and controls had comparable age (p = 0.49) and gender distribution (p = 0.74).

### Vaccine

To immunize children, a national drug (i.e., a purified adsorbed diphtheria-tetanus toxoid with a reduced antigen level [ADS-m toxoid]) produced by Biomed JSC named for I. I. Mechnikov (Russia) was used. The vaccination dose (0.5 ml) contained 5 flocculating units (LF) of diphtheria and 5 antitoxin-binding units (EC) of tetanus toxoid. Children were not revaccinated against pertussis, since combined vaccines with an acellular pertussis component have not been registered in our country. Moreover, a DTaP vaccine containing a whole cell component is indicated only for children under 4 years old in Russia.

Children were revaccinated with the ADS-m toxoid (0.5 ml, i/m, into the shoulder deltoid) once.

### Exclusion Criteria

Children received a full primary course of vaccination and revaccination (4 doses of any vaccines against diphtheria, tetanus, or in combination with pertussis before the diagnosis); children vaccinated against diphtheria, tetanus, or pertussis within the last 2 years after a carcinoma treatment; allergy or hypersensitivity to any vaccine component including previous reactions to the vaccine; acute respiratory diseases within the last 2 months; daily intake of oral nonsteroidal anti-inflammatory drugs; administration of blood products or immunoglobulins within the last 3 months; immunodeficiencies, metastasis, neoplasms within the last 2 years after the treatment; or neurological disorders.

## Methods

### Clinical Safety of the Vaccination

Drug safety was assessed by individual examination and questioning of all the vaccinated patients, as well as recording of local and systemic reactions to the ADS-m toxoid within 14 days and 1 month after the vaccination.

### Isolation of Leukocytes

Peripheral blood mononuclear leukocytes (PBMLs) were isolated from whole blood in a ficoll-urografin density gradient [10^6^ cells per 1 ml of RPMI-1640 medium (PanEko, Russia)].

### Subpopulation of Lymphocytes

Subpopulations of peripheral blood lymphocytes were investigated *in vitro* by flow cytometry with Cytomix FC-500 (Beckman Coulter, USA) using monoclonal antibodies (mAbs) to CD3-FITC/CD16/56-PE, CD45-FITC/CD3-ECD/CD20-PE, CD45-FITC/CD3-PE/CD4-PC5, and CD45-FITC/CD3-PE/CD8-PC5 (Immunotech, France**).** As a comparison, we used pediatric age-specific standards obtained in the clinical and immunological laboratory of the Research Institute of Children Oncology and Hematology Federal State Budgetary Institution Blokhin National Medical Research Center of Oncology of the Ministry of Healthcare of the Russian Federation and the Federal State Budgetary Institution State Scientific Center Institute of Immunology of the FMBA of the Russian Federation.

### Determination of Diphtheria and Tetanus Toxoid IgG Concentrations

The serum level of specific antibodies to diphtheria and tetanus was investigated by enzyme-linked immunosorbent assay (ELISA) using commercial kits designed as assays for IgG antibodies (Euroimmun AG, Germany) (anti-diphtheria toxoid (*Anti-Diphtheria Toxoid* ELISA, IgG) and anti-tetanus toxoid (*Anti-Tetanus toxoid* ELISA, IgG). According to the manufacturer’s instructions, reference values of anti-diphtheria immunity were as follows: <0.1 IU/ml - negative; 0.1-1.0 - short-term protection, boosting is required; > 1.0 - positive. As for anti-tetanus immunity, reference values were as follows: <0.01 IU/ml - negative; 0.01-0.1 - doubtful; 0.1-0.5 - short-term protection, boosting is required; > 0.5 - positive.

### Statistical Methods

The data check for compliance with the normal distribution law (Shapiro-Wilk test) showed that distribution of most of the investigated parameters was different from the normal one. Descriptive statistics of absolute and percentage of certain lymphocyte types were presented by the median and interquartile range [Me(Q1-Q3)]. Descriptive statistics of the level of IgG antibodies were presented by the geometric mean and 95% confidence interval [GMC (95% CI)]. The analysis of repeated measurements of quantitative parameters at control points of the study was carried out with Friedman’s nonparametric one-way analysis of variance for linked samples. Multiple post-hoc comparisons of the values with the pre-revaccination level at the control points were performed with the Demsar method. Quantitative indicators of the study groups were compared with the Mann-Whitney test. The median value was compared with the normal range with the Wilcoxon signed rank test. Qualitative nominal indicators of independent samples were compared with the Pearson Chi-square test during an analysis of contingency tables. Differences were considered statistically significant at p ≤ 0.05. All the calculations were performed in the free R statistical environment (v.3.6, GNU GPL2 license).

## Results

### Post-Vaccination Clinical Course

Twenty-six children were revaccinated with the ADS-m toxoid. Early (<30 days) and late (<12 months) post-vaccination periods were uneventful in most children. During the early post-vaccination period, two (7.7%) children showed vaccination reactions (hyperemia [>5 cm] in the injection site for 3-4 days; an increased body temperature to 38.0°C on the first day).

At 6.5 months after the immunization, one child underwent a surgical intervention to manage adhesive intestinal obstruction. At 10 months after the vaccination, an increase in blood pressure of unknown origin was detected in one child. At 1.5 years after the immunization, a post-surgical disturbance of intestinal motility associated with fecal impaction in the large intestine was mentioned in one girl. After treatment by a neuropsychiatrist, the child’s condition improved. No abnormal events in these children can be attributed to late reactions to the immunization.

It is noteworthy that a follow-up examination including an oncological examination, as well as additional tests in accordance with a nosological form of the neoplastic process did not reveal any signs of recurrence and metastasis both in revaccinated children and controls without revaccination within a year after the immunization.

### Post-Vaccination Antibody Level

**Figure 1:**
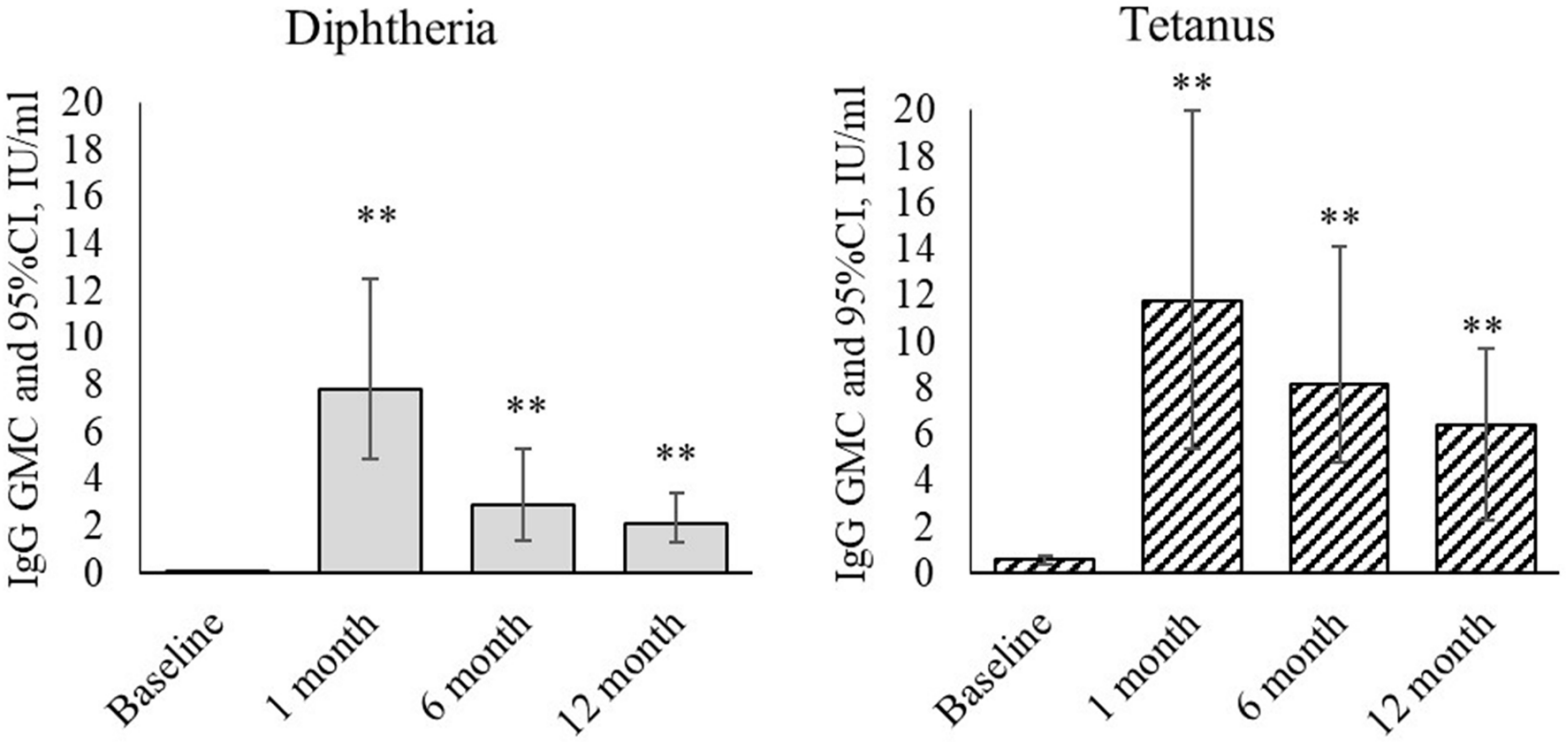
Dynamics of IgG antibodies against diphtheria and tetanus during revaccination with ADS-m toxoid (n = 26). Geometric mean concentrations and their 95% confidence interval (GMC [95% CI]). **p < 0.001 - differences from the pre-revaccination value (we used the post-hoc Demsar test with a statistically significant Friedman omnibus test).

See the level of IgG antibodies against diphtheria and tetanus (ELISA) in **Figure 1**.

Revaccination resulted in statistically significant changes in concentration of IgG antibodies against diphtheria (p <0.001) and tetanus (p <0.001). At a month after the revaccination, the geometric mean concentration of IgG antibodies against diphtheria increased from 0.02 [0.003-0.044] IU/ml to 7.8 [4.9-12.5] IU/ml (p <0.001), and the value against tetanus increased from 0.59 [0.41-0.74] IU/ml to 11.8 [5.4-20.0] IU/ml (p <0.001). At 6 months after the revaccination, the IgG antibody level was lower than in 1 month after it. However, their geometric mean concentration remained statistically significantly higher than before the revaccination [2.9 (1.4-5.3) IU/ml - antibodies against diphtheria (p <0.001), and 8.2 (4.8-14.1) IU/ml - antibodies against tetanus (p <0.001)]. At 12 months after the revaccination, the IgG antibody level remained statistically significantly higher than before the revaccination. The geometric mean concentration was 2.1 [1.3-3.4] IU/ml (antibodies against diphtheria) (p <0.001) and 6.4 [2.3- 9.7] IU/ml (antibodies against tetanus) (p <0.001).

### Subpopulation of Lymphocytes During the Revaccination

**Table 2** illustrates lymphocyte alterations detected in 41 children (26 revaccinated ones and 15 controls) with a history of solid tumors as compared to healthy controls.

**Table 2:** Comparative characteristics of peripheral blood lymphocyte subpopulations in children with a history of solid tumors and healthy ones aged 7-15 years.

Parameters	Healthy children (normal range)	Patients^1^			p^3^ (including the normal range)	
		Vaccine (N=26)	Controls (N=15)	p^2^	Vaccine	Controls
Leukocytes	6,000 (5,024-6,970)	8,014 (6,220-9,795)	8,588 (6,270-10,202)	p=0.95	p=0.001	p=0.009
Lymphocytes, %	39.5 (35-43)	32 (27-37)	33.5 (28-38)	p=0.45	p<0.001	p=0.004
Lymphocytes, abs.	3,500 (2,350-4,620)	2,490 (1,908-3,119)	2,544 (1,695-3,115)	p=0.8	p<0.001	p=0.001
Т lymphocytes (CD45/СD3+), %	71 (65-78)	68 (60-74)	68 (64-71)	p=0.47	p=0.002	p=0.04
Т lymphocytes (CD45/СD3+), abs.	1,700 (1,312-2,028)	1,682 (1,259-2,195)	1,682 (1,259-2,195)	p=0.69	p=0.95	p=0.57
T helpers (CD45/CD3/СD4+), %	37 (32-41)	41 (34-47)	41 (34-54)	p=0.59	p=0.07	p=0.14
T helpers (CD45/CD3/СD4+), abs	900 (750-1,100)	1,049 (712-1,268)	1,042 (849-1,313)	p=0.72	p=0.11	p=0.07
Cytotoxic Т lymphocytes, (CD45/CD3/СD8+), %	31 (25-34)	25 (22-27)	24 (20-29)	p=0.79	p<0.001	p=0.004
Cytotoxic Т lymphocytes, (CD45/CD3/СD8+), abs	750 (601-854)	612 (435-766)	615 (396-788)	p=0.86	p=0.002	p=0.03
Natural killer cells, NK cells (CD16/56+), %	12.5 (7-18)	14 (12-17)	15 (10-17)	p=0.82	p=0.02	p=0.05
Natural killer cells, NK cells (CD16/56+), abs	250 (210-316)	328.8 (249-416)	333 (266-406)	p=0.86	p=0.002	p=0.009
B cells (CD45/CD20+), %	12.5 (8-16)	10 (7-12)	10 (7-14)	p=0.97	p<0.001	p=0.02
B cells (CD45/CD20+), abs	400 (325-494)	267.7 (213-299)	262 (204-304)	p=0.97	p<0.001	p=0.001
CD4/CD8 IRI	1.25 (0.8-1.7)	1.7 (1.3-2.1)	1.6 (1.3-2)	p=0.49	p<0.001	p=0.005

^1^medians and interquartile range: Me (Q1-Q3).

^2^patient groups were compared with the Mann-Whitney U-test.

^3^comparison with the normal range was carried out with the Wilcoxon signed rank test.

As compared to the normal range, the group of children with a history of solid tumors demonstrated a statistically significant increase in the number of leukocytes, natural killer cells (absolute and relative values), and immunoregulatory index (IRI). At the same time, absolute and relative numbers of lymphocytes, cytotoxic T lymphocytes, and B cells, as well as the relative number of T lymphocytes were statistically significantly lower than normal ones. These patterns were similar for both the revaccinated and not revaccinated children. These groups did not differ from each other before the revaccination.

The analysis of subpopulations of peripheral blood lymphocytes in children with a history of solid tumors not revaccinated with the ADS-m toxoid (n = 15) (control group) did not reveal any significant in-examination differences in comparison with the baseline values. This means that the cell pool did not recover in unvaccinated persons over time (follow-up period was 12 months). At the same time, vaccinated patients showed a statistically significant change in the number of parameters, as compared with the baseline, at the control points.

The post-revaccination absolute number of lymphocytes (**Figure 2A**) tended to increase without statistically significant differences (p = 0.38) [i.e., from 2,490 (1,908-3,119) to 2,978 (2,245-3,809)] after 12 months. Along with this, post-revaccination lymphocyte percentage significantly changed (p = 0.03) increasing from 32 (26.5-37)% to 39 (31.5-44)% after 12 months (p = 0.02). As a result of the growth, lymphocyte percentage in revaccinated children became statistically significantly higher than in controls (p = 0.05) at 12 months after the vaccination.

**Figure 2A:**
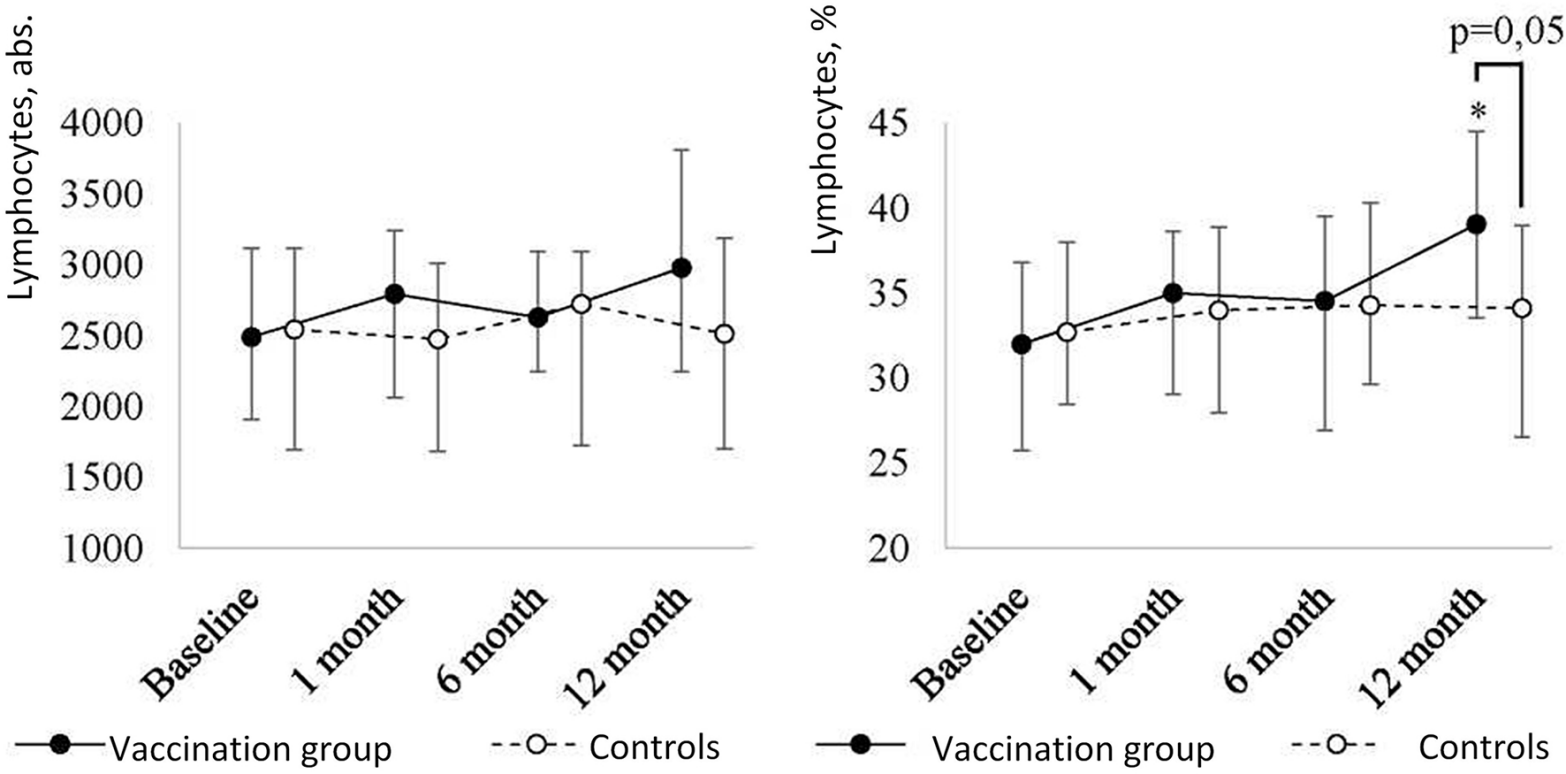
Peripheral blood lymphocyte level dynamics in children with a history of solid tumors in the study group (after revaccination with the ADS-m toxoid, n = 26) and the control group (n = 15), the median and the interquartile range are shown [Me (Q1-Q3)]. *statistically significantly different from the pre-vaccination value (p ≤ 0.05). To analyze alterations, the post-hoc Demsar test was used with a statistically significant Friedman omnibus test. Study groups were compared with the Mann-Whitney test.

At one month after the revaccination, further analysis of the lymphocyte pool (**Figure 2B**) revealed a statistically significant increase in the absolute and relative number of T lymphocytes (CD45/CD3+) (p = 0.04 and p = 0.007, respectively) with a further decrease to the baseline in revaccinated patients.

**Figure 2B:**
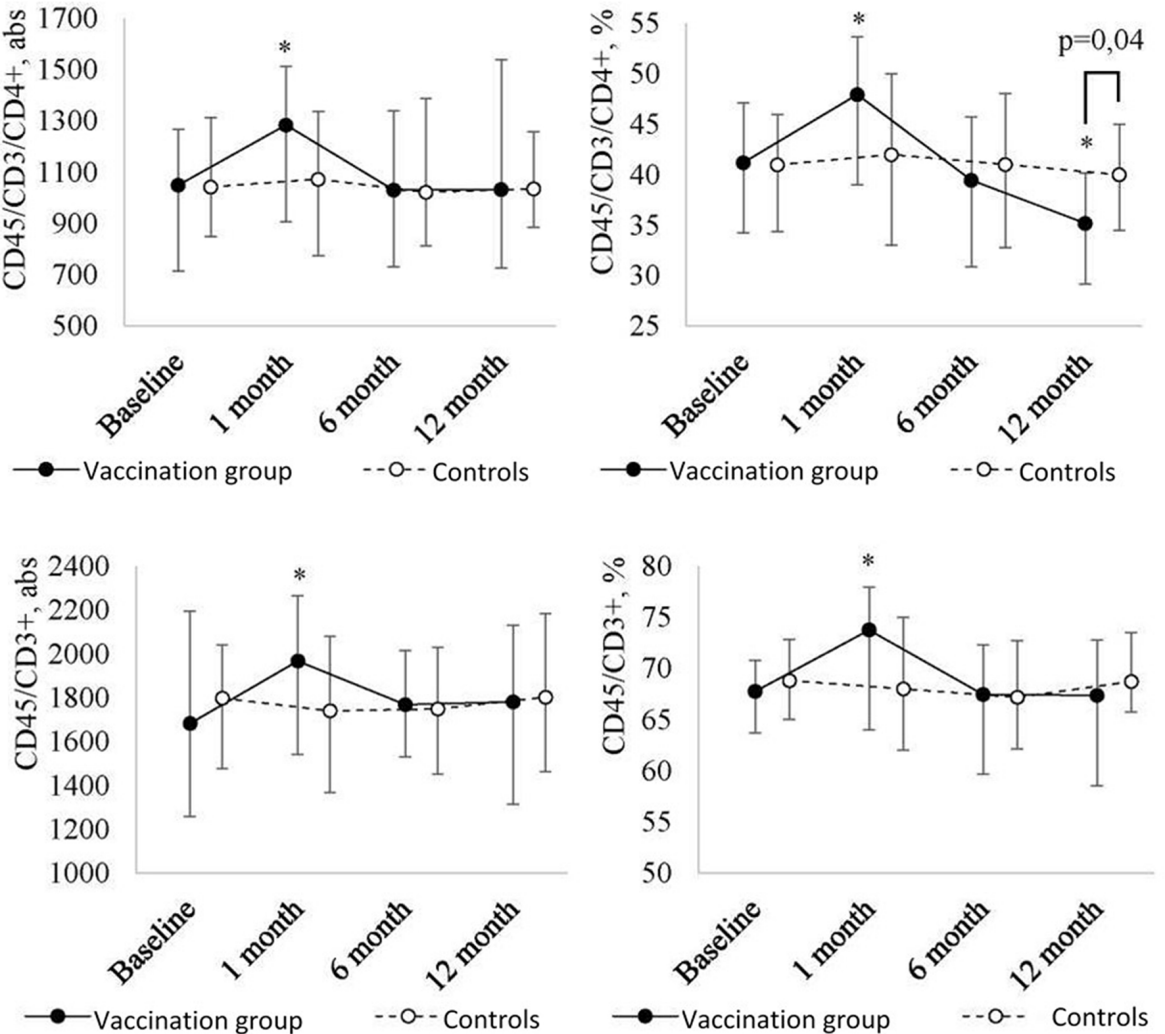
Peripheral blood T lymphocyte (CD45/СD3+, CD45/CD3/СD4+) level dynamics in children with a history of solid tumors in the study group (after the revaccination with the ADS-m toxoid, n = 26) and the control group (n = 15), the median and the interquartile range are shown [Me (Q1-Q3)]. *statistically significantly different from the pre-vaccination value (p ≤ 0.05). To analyze alterations, the post-hoc Demsar test was used with a statistically significant Friedman omnibus test. Study groups were compared with the Mann-Whitney test.

As for T helpers (CD45/CD3/CD4+), the pattern was as follows. Within the first month after the vaccination, their level significantly [p = 0.04 (abs) and p = 0.02 (%)] increased [48 (39-54)%] as compared with the baseline [41 (34-47)%]. After 6 months, it returned [39 (31-46)%] to the baseline value. After 12 months, it was lower than the baseline (p = 0.004). As a result of the decrease, the relative number of T helpers in revaccinated patients became statistically significantly lower than in controls (p = 0.04) (35 (29-41)% *vs*. 40 (35-53)%) at 12 months after the vaccination.

In addition, the revaccination with the ADS-m toxoid led to a statistically significant change in absolute and relative numbers of NK cells (CD16/56+) (p = 0.009 and p <0.001, respectively) and B cells (CD45/CD20+) (respectively, p = 0.005 and p <0.001) (**Figure 2C**). At 12 months after the revaccination, a statistically significant decrease in absolute number of NK cells (CD16/56+) (p = 0.006; from 328 (249-416) to 207 (97-260)) and B cells (CD45/CD20+) [p = 0.05; from 257 (213-299) to 219 (115.5-260)] was detected. Relative number of NK cells (CD16/56+) and B cells (CD45/CD20+) decreased from 14.5 (12-17)% to 8.5 (5.5-12)% (p <0.001), and from 10 (7-12)% to 6 (4-8.5)% (p <0.001), respectively. It should be noted that a decreased level of NK cells was observed throughout the post-vaccination period. In relative terms, a statistically significant decrease was noted already at 1 month after the revaccination (p = 0.01), in absolute terms, it was detected in 6 months (p = 0.03). As a result of the decrease, NK cell level in revaccinated patients became lower than control values at 1 month (relative number) and 6 months (absolute number) after the vaccination.

**Figure 2C:**
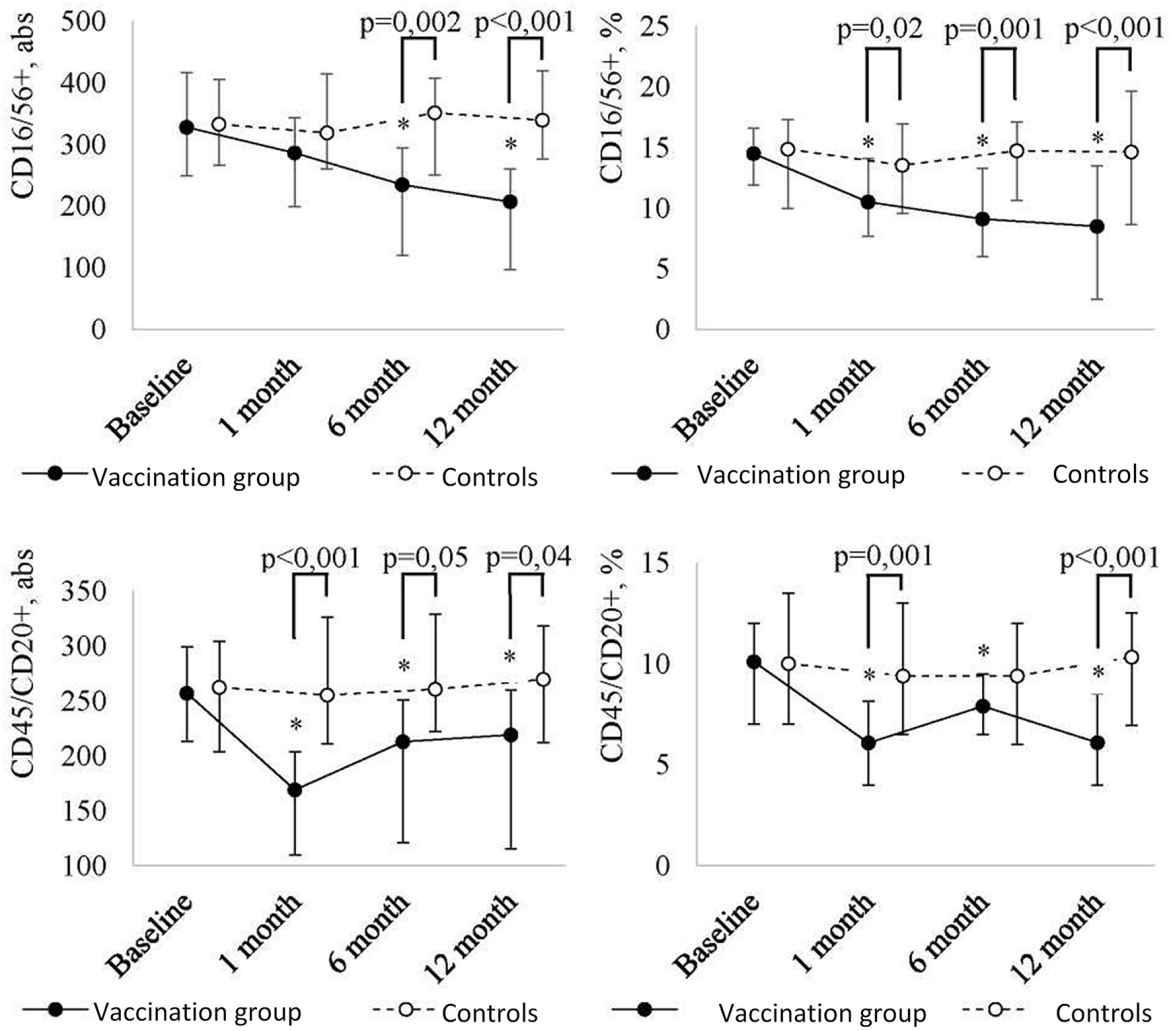
Peripheral blood NK cell (CD16/56+) and B cell (CD45/CD20+) level dynamics in children with a history of solid tumors in the study group (after the revaccination with the ADS-m toxoid, n = 26) and the control group (n = 15), the median and the interquartile range are shown [Me (Q1-Q3)]. *statistically significantly different from the pre-vaccination value (p ≤ 0.05). To analyze alterations, the post-hoc Demsar test was used with a statistically significant Friedman omnibus test. Study groups were compared with the Mann-Whitney test.

The most intense decrease in B cells (CD45/CD20+) followed by stabilization of the value below the baseline was observed at 1 month after the revaccination (p <0.001 [abs] and p = 0.002 [%]). The difference between the revaccinated and the control group was evident even 1 month after the vaccination, and it persisted for ≤12 months.

## Discussion

In the present paper, it was of interest to study the effect of vaccines against diphtheria and tetanus on immune response formation in children with prior malignant neoplasms during prolonged (more than 2 years) remission.

There is a certain approach to immunizing children with a history of oncological diseases who receive specific antineoplastic treatment. It lies in the fact that inactivated polysaccharide vaccines and toxoids should be administered at ≥3 months after therapy withdrawal (7).

Our results indicate that the post-vaccination period is favorable in such children. We did not notice any strong or unusual reactions to the ADS-M vaccine. It should be noted that children underwent a medical checkup by an oncologist, as well as a number of clinical, laboratory, and instrumental tests before the vaccination. Within a year (i.e., a follow-up period), the vaccinated children showed no signs of recurrent underlying disease or metastasis. Thus, the study confirms the safety data of diphtheria and tetanus vaccine prophylaxis in children with a history of solid tumors during prolonged remission.

It is known that immunization must not cause pronounced changes in the functional activity of a child’s immune system beyond physiological reaction limits. The comparative analysis showed that, in contrast to healthy children, those with a history of cancer demonstrated cellular immunity deviations. In general, they represented a decrease in the relative number of mature T lymphocytes, as well as the absolute and relative number of cytotoxic T cells and B lymphocytes. Lack of T cell immunity can be induced by a prior neoplastic process and therapeutic interventions representing a stress factor for hematopoietic cells (for example, chemotherapy, radiation therapy, infection, and transplantation). In such cases, outcomes depend on the immune system’s restoration degree (especially, T lymphocytes) (8). It is not inconceivable that an immune deficiency background could be a tumorigenic factor.

It was shown that, despite a decrease in the number of B lymphocytes at a month after the revaccination with the ADS-m toxoid, their functional activity was preserved providing protective levels of antibodies against diphtheria and tetanus. Perhaps a decreased number of B lymphocytes is associated with their pool depletion after the antigenic load. A number of other alterations (i.e., an increase in the absolute and relative number of mature T lymphocytes and T helpers at 1 month after the revaccination) were transient and reversible. The parameter values recovered after 6-12 months. A persistent cryptogenic decrease in the absolute and relative numbers of natural killer cells was reported in 1, 6, and 12 months after the revaccination. Probably, the event can result from extreme susceptibility of young regenerating cells to homeostatic alterations induced by microbial and viral antigens, and these cells may become apoptotic more often than mature immunocytes. It should be noted that the dynamics of relative values were more significant than in absolute values. In this regard, we suggest that evaluation of cellular immunity parameter alterations should be focused on their relative values.

The ability of T cells to proliferate, to accumulate, and to maintain an efficient response to a wide range of antigens depends on a repertoire of unique T cell receptors (TCR) produced by the thymus during the development of T lymphocytes. The process depends on cross-interactions between bone marrow precursors of T cells supporting the microenvironment of the thymic stroma which, mainly, consists of epithelial, endothelial, and mesenchymal stromal cells, as well as dendritic cells and macrophages (8–10). Nevertheless, for example, IL-7- and IL-15-driven T cell proliferation induced by lymphopenia can promote the numerical recovery of T cells. Impaired quantitative and functional recovery of T cells (in particular, CD4+ T lymphocytes (11–14) was directly associated with an increased risk of opportunistic infections (12, 15, 16), relapses (17), and overall unfavorable clinical outcomes (18, 19).

In contrast to the relatively early recovery of innate immunity cells, hematopoietic stem cell recipients and patients with solid tumors have a long-term deficiency of T lymphocytes and B cells whose full recovery can take more than 2 years (8). In our paper, the revaccination against diphtheria and tetanus, in general, promoted proliferation of the total lymphocytic cell pool along with restoration of T lymphocyte subpopulations in children with a history of solid tumors. It can be assumed that the vaccination had a certain nonspecific immunomodulatory effect because the vaccine against diphtheria and tetanus includes toxoids of the same bacteria, which can target polarization of the immune response along the Th-1 pathway. At the same time, it is necessary to consider adjuvant properties of aluminum compounds (i.e., ADS-m ingredients) which are mediated by several mechanisms such as depot effect, stimulation of antigen (AG) phagocytosis, activation and maturation of antigen-presenting cells (APC), complement activation, and stimulation and differentiation of CD4+ T cells (20, 21).

A fundamentally important result of the study was the assessment of ADS-m immunogenicity (i.e., the ability of the immune system to develop specific immunity, to have a boosting effect,and to preserve specific antibodies for a long time during disease remission) in children with a history of solid tumors.

We noted that children who, mainly, did not complete a primary immunization course before a neoplastic diagnosis can maintain sufficient levels of specific antibodies against tetanus (0.59 IU/ml; reference values > 0.5 are positive), despite the disease and received antineoplastic treatment, for 2 or more years after the vaccination. However, several individuals may lose existing specific immunity. In our paper, this was noted in relation to anti-diphtheria antibodies that may be associated with intensive immunosuppressive therapy. Other authors obtained similar results (22). However, previous studies showed that it was safe to administer inactivated vaccines to such pediatric populations, and they responded to the vaccination as actively as healthy children (22–25). Revaccination of children with a drug containing reduced amounts of diphtheria (5 LF) and of the tetanus toxoid (5 EC) (ADS-m), despite a long interval from the moment of primary vaccination, led to intense specific antitoxic immunity against diphtheria. At 1 month after the revaccination, the geometric mean concentration of IgG antibodies increased from 0.02 IU/ml to 7.8 IU/ml (p <0.001), and the geometric mean concentration of IgG antibodies against tetanus increased from 0.59 IU/ml to 11.8 IU/ml (p <0.001). After 12 months, the value remained quite high. This indicates that children with a history of carcinoma maintain immunological memory for a long time, and they do not lose an ability to form specific immunity. Antibodies are produced, and a sufficiently high level persists for a long time.

## Conclusion

During remission of solid tumors, children demonstrate a decrease in the number of T lymphocytes, cytotoxic T cells, and B lymphocytes. Revaccination with ADS-m toxoids led to a short-term increase in the number of T cells, and the number of CD4 + T cells after 12 months was still lower compared to those vaccinated. Despite the lack of dynamics in replenishing the B lymphocyte population, they retained a high potential to produce antibodies. Thus, it can be assumed that the vaccine has a specific effect against diphtheria and tetanus, as well as an immunomodulatory effect towards T cells. So, the immune response can be cell-mediated which is important to prevent both a homonymous infection and neoplastic recurrence, as well as to protect from viruses. The issue seems to be especially relevant during the SARS-CoV-2 pandemic.

Revaccination with the ADS-m toxoid is accompanied by production of antibodies against diphtheria and tetanus at high protective levels, and this does not cause disease recurrence and metastasis in children with a history of solid tumors during the follow-up period.

## Data Availability Statement

The raw data supporting the conclusions of this article will be made available by the authors, without undue reservation.

## Ethics Statement

The studies involving human participants were reviewed and approved by Ethics Committee of the Federal State Budgetary Institution Blokhin National Medical Research Center of Oncology of the Ministry of Healthcare of the Russian Federation. Written informed consent to participate in this study was provided by the participants’ legal guardian/next of kin.

## Author Contributions

MK - head of the project, description of the results. NA - carrying out immunological research methods. SK - collection of biological materials, clinical characteristics of patients, observation of vaccinated patients. AV - statistical data processing. VP - vaccination and evaluation of the post-vaccination period. AK - review of recent research, design and translation of published materials. All authors contributed to the article and approved the submitted version.

## Funding

The work (research and publication fees) was funded from the personal funds of the authors.

## Conflict of Interest

The authors declare that the research was conducted in the absence of any commercial or financial relationships that could be construed as a potential conflict of interest.
